# Multiple Bacteria Identification in the Point-of-Care: an Old Method Serving a New Approach

**DOI:** 10.3390/s20123351

**Published:** 2020-06-12

**Authors:** Sara Viveiros, Mónica Rodrigues, Débora Albuquerque, Sofia A. M. Martins, Susana Cardoso, Verónica C. Martins

**Affiliations:** 1 Instituto Superior Técnico, University of Lisbon, 1049-001 Lisboa, Portugal; sara.viveiros@tecnico.ulisboa.pt (S.V.); debora.albuquerque@ist.utl.pt (D.A.); susana.freitas@tecnico.ulisboa.pt (S.C.); 2 INESC-MN- Microsystems and Nanotechnologies, 1000-029 Lisboa, Portugal; smartins@inesc-mn.pt; 3 Centre for Ecology, Evolution and Environmental Changes, Faculdade de Ciências, Universidade de Lisboa, 1749-016 Lisboa, Portugal; marodrigues@fc.ul.pt

**Keywords:** magnetoresistance, spin-valve, asymmetric PCR, biosensor, bacterial pathogens

## Abstract

The accurate diagnosis of bacterial infections is of critical importance for effective treatment decisions. Due to the multietiologic nature of most infectious diseases, multiplex assays are essential for diagnostics. However, multiplexability in nucleic acid amplification-based methods commonly resorts to multiple primers and/or multiple reaction chambers, which increases analysis cost and complexity. Herein, a polymerase chain reaction (PCR) offer method based on a universal pair of primers and an array of specific oligonucleotide probes was developed through the analysis of the bacterial 16S ribosomal RNA gene. The detection system consisted of DNA hybridization over an array of magnetoresistive sensors in a microfabricated biochip coupled to an electronic reader. Immobilized probes interrogated single-stranded biotinylated amplicons and were obtained using asymmetric PCR. Moreover, they were magnetically labelled with streptavidin-coated superparamagnetic nanoparticles. The benchmarking of the system was demonstrated to detect five major bovine mastitis-causing pathogens: *Escherichia coli*, *Klebsiella* sp., *Staphylococcus aureus*, *Streptococcus uberis,* and *Streptococcus agalactiae*. All selected probes proved to specifically detect their respective amplicon without significant cross reactivity. A calibration curve was performed for *S. agalactiae,* which demonstrates demonstrating a limit of detection below 30 fg/µL. Thus, a sensitive and specific multiplex detection assay was established, demonstrating its potential as a bioanalytical device for point-of-care applications.

## 1. Introduction

Bacterial infections are considered one of the main sources of disease worldwide. Bovine mastitis is a widespread disease in modern dairy herds, characterized by the inflammation of the mammary gland. It can be caused by a multitude of different pathogens, which have a significant impact in the dairy industry, mainly due to milk losses, poor milk quality, and culling of animals [1]. The main mastitis-causing pathogens are *Escherichia coli*, *Streptococcus uberis,* and *Staphylococcus aureus*, as well as a wide variety of other organisms that have been identified as potential mastitis pathogens. These organisms are termed major pathogens and are generally regarded as those commonly associated with clinical mastitis in dairy cattle. Mastitis prevention and treatment consists in the regular administration of antibiotics, even though this entails high treatment costs and poor efficacy, also posing serious risks in the emergence of bacterial antimicrobial antibiotic resistance (AMR), which has become a serious public health concern. The standard diagnosis methods for bacterial identification involve microbial culture which, although accurate in identifying disease, the respective infectious agents and antibiotic resistant phenotypes is a slow process and demands specialized facilities and personnel [2]. It also should be noted that sample collection, conditioning, and transportation to centralized laboratories delays test results and treatment decisions. Thus, there is still a need for a diagnostic device capable of providing quick, accurate, and on-site diagnosis of the disease, which will thus contribute to a more effective and targeted treatment.

Biomolecular approaches based on nucleic-acid amplification such as polymerase chain reaction (PCR) offer significant advantages over conventional culture plate methods in terms of accuracy and turnaround time [3]. Additionally, PCR-based assays are able to detect growth-inhibited bacteria, leading to a significant decrease in false negative results, which have been reported to occur with a probability of 27–50% with culture plate methods [4]. However, results still depend mostly on experts and dedicated facilities. Therefore, considerable solutions have been focusing on the miniaturization of these biomolecular techniques into new, easy-to-use, point-of-care (POC) devices through the integration of microfluidic systems, which is a crucial element to minimize user intervention and automate biological experiments [5]. In comparison to microbial culture, POC PCR-based systems are expected to bring improvements in terms of time of analysis, sample volume, and the ability to analyze multiple targets in the same assay. While bacterial culturing-based detection of pathogens requires sample incubation for at least 18 h [6], result times can be significantly reduced to a couple of hours using fully integrated systems that comprise miniaturized sample preparation and amplification units [2].

In a POC device, detection of amplified sequences is often based on DNA hybridization with complementary immobilized probes, which can be spotted in planar surfaces in a microarray-like format. The resource to several highly specific probes, complementary to different targets, can significantly enhance the multiplex capability of diagnostic devices, enabling to rule out different infectious agents in a single assay [7]. However, multiplex amplification protocols can be particularly challenging at the microscale in microfluidic settings. To apply a multiplex amplification assay in a POC device, there are two possible approaches, both of which present limitations. On one hand, parallel singleplex reactions in multiple reaction chambers is limited by the dimensions and complexity of the microfluidic device, which only allows for the amplification of a few target sequences. On the other hand, the amplification of various target sequences in the same reaction chamber by multiple pairs of primers [8] tends to suffer from poor sensitivity, due to different amplification efficiencies and preferential production of one target over the others [9].

A possible alternative to tackling this challenge may lay in the use of a single pair of primers able to indiscriminately amplify any target bacteria. To achieve this, a conserved sequence in the bacterial genome is required. Over the past few decades, the 16S ribosomal RNA (rRNA) gene has been used as a molecular marker to identify specific microbial taxa and their phylogenetic classification [10,11,12,13]. It is present in all prokaryotic cells and has conserved variable sequence regions that allow for the use of a simultaneous universal amplification, as well as the identification of close relationships at the genus or the differentiation at the species level [10]. Thus, the conserved regions of this gene can be used to design a pair of primers that targets interspecific regions of the 16S rRNA gene, further allowing for the design of a specific oligonucleotide probe that can be used in a POC device for bacteria identification. This approach represents a practical alternative to the use of several PCR reaction chambers or multiple pairs of primers in the amplification reaction mixture.

In this work, an existing magnetoresistive (MR) biochip and respective portable electronic reader [14,15,16] were applied in the genetic analysis of the 16S rRNA gene for the multiplex detection of five major mastitis-causing bacteria. The MR biochip was comprised of an array of 30 magnetic field sensors (spin-valves) that allowed for multiple probe immobilization. Spin-valves offer particular advantages in terms of sensitivity, low limit of detection, high signal to noise ratio, small size, and integration possibility [17,18]. In a magnetic field biosensor, the target analytes are labelled with magnetic nanoparticles (MNPs), which generate a fringe field when exposed to an external magnetic field, promoting a proportional change in the sensor’s electrical resistance [19,20]. Here, oligonucleotide probes were designed to specifically target each bacteria based on conserved regions of the 16S rRNA gene that, nevertheless, contained sequence differences between species. The sensitivity and cross reactivity of this multiplex system was evaluated by detection assays with targets amplified by asymmetric PCR using one pair of universal primers. 

## 2. Materials and Methods

### 2.1. Sensor and Chip Microfabrication

**Figure 1:**
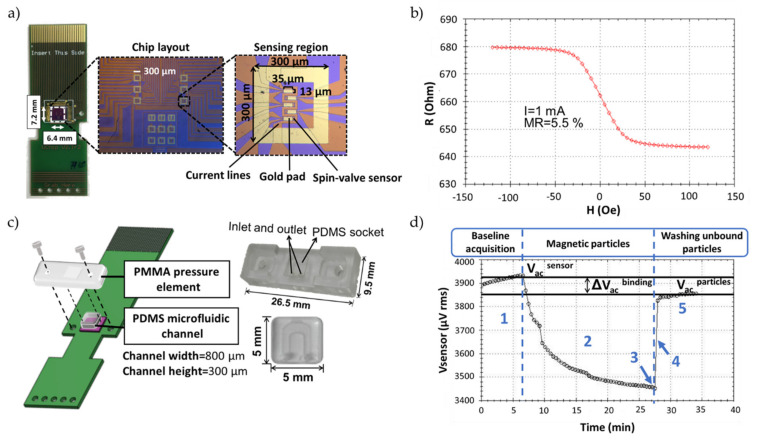
(**a**) Biochip (7.2 × 6.4 mm^2^) encapsulated in a printed circuit board (PCB) chip carrier. (**b**) Transfer curve of a U-shaped spin-valve sensor (80 × 2.6 µm^2^). (**c**) Schematic representation of the microfluidic system elements, showing how the alignment is achieved. Sealing of the polydimethylsiloxane (PDMS) channel is achieved by using a screw in each end of the polymethylmethacrylate (PMMA) pressure element. (**d**) Voltage signal acquired from one spin-valve sensor during the detection of a complementary DNA target hybridization event labeled with 250 nm magnetic particles. A measurement comprises five phases: 1) acquisition of the sensor baseline signal (V_ac_^sensor^); 2) decreasing signal due to the magnetic particles settling down over the sensor; 3) saturation signal when all particles have settled; 4) washing steps with wash off of all the unbound particles; 5) final signal corresponding to the presence of target bound magnetic particles over the sensor (V_ac_^particles^).

The biochip consists of an array of 30 spin valve (SV) sensors passivated with an oxide layer, arranged in six sensing regions, each one containing five active sensors covered with a gold layer (35 × 13 µm^2^), and surrounded by a gold frame for discrete spotting of the probes (see Figure 1a). The fabrication of the SV biochips entails several microfabrication steps [21]. Briefly, a 6-inch silicon wafer passivated with 50 nm of alumina (Al_2_O_3_) is used as a substrate and the SV metallic multi-layer structure is deposited by ion beam deposition, consisting of Ta 20 nm/NiFe 25 nm/CoFe 28 nm/Cu 26 nm/CoFe 24 nm/MnIr 70 nm/Ta 50 nm. The sensors are defined by direct write laser photolithography and ion milling, arranged in series of two sensors (active area of 80 × 2.6 µm^2^) electrically contacted by aluminum leads. The sensors’ average magnetoresistance is 6.0% and sensitivity of 1.3%/mT (see Figure 1b).

### 2.2. Biochemical Reagents

The water used in the preparation of all solutions was ultra-pure. When not disclosed, reagents are from Fischer Scientific. A TRIS-EDTA (TE) buffer was prepared by combining TRIS, ethylenediaminetetraacetic acid (EDTA), and K_2_HPO_4_ at 10 mM, 1 mM, and 100 mM concentration, respectively. pH was adjusted to 7.4 using 1 M HCl. A TRIS-borate-EDTA (TBE) buffer 10x was acquired from FRILABO (Porto, Portugal). Phosphate buffer (PB) 0.1 M, pH 7.2 was prepared from stock solutions of Na_2_HPO_4_ and NaH_2_PO_4_ at 0.2 M. PB-Tween20 consisted on PB buffer with 0.02% (v/v) of Tween^®^ 20 from Promega (Madison, WI, USA).

The customized single stranded oligonucleotide sequences were synthesized by STABVIDA (Caparica, Portugal).

DNA-free water, MasterMix 16S Basic, and Moltaq 16S from Molzym (Germany) were used for PCR reactions. Electrophoresis reagents included TopVision agarose from Thermo Fisher Scientific^TM^ (MA, USA), for gel preparation, GRS DNA loading buffer blue 6x from GRiSP (Porto, Portugal), GeneRuler 1 kb DNA ladder from Thermo Fisher Scientific^TM^, and Diamond^TM^ nucleic acid dye from Promega.

The MNPs were nanomag^®^-D from Micromod (Rostock, Germany), with a diameter of 250 nm and 75–80% (w/w) magnetite in a matrix of dextran (40 kDa), streptavidin coated. The particles have a magnetic moment of ∼1.6 × 10^−16^ Am^2^ for a 1.2 kA/m magnetizing field and a susceptibility of *χ ∼* 4.

### 2.3. Bacterial Samples

Bacterial isolates of *Escherichia coli*, *Klebsiella* species, *Staphylococcus aureus*, *Streptococcus uberis,* and *Streptococcus agalactiae* were obtained from mastitis samples and provided by Dr. Ricardo Bexiga from Faculdade de Medicina Veterinária (FMV, Lisbon University). Bacterial identification was performed by colony observation after grown in selective media and when necessary, further evaluated by analytical profile index (API) assays. All bacterial strains were grown at 37 °C on brain heart infusion (BHI) agar plates.

DNA was extracted from bacterial cultures using Qiagen DNeasy Blood and Tissue kit (Life Technologies, CA, USA), according to supplier’s instructions. Genomic DNA was quantified using Nanodrop 2000C spectrophotometer (Thermo Fisher Scientific, Waltham, MA, USA) and stored at 20 °C until further use.

### 2.4. Probe and Primer Sequence Design

Probe and primer design was performed using 16S rRNA gene sequences representative of the most common bacteria known to cause mastitis (e.g., *S. aureus*, *S. epidermidis*, *S. agalactiae*, *S. uberis*, *Klebsiella* sp, *E. coli*). All sequences were obtained from the GenBank database and multiple alignments were constructed using SEQUENCHER (Gene Codes Corporation, MI, USA), as well as the ClustalW multiple sequence aligner [22]. The conserved regions were chosen to design universal primer pairs, which delimit a 700 bp sequence. The forward primer is biotinylated on the 5’ end. Primer’s sequences and characteristics are summarized in Table 1. For the identification of each species, a probe was designed for each bacteria when a conserved region within the species was identified and the region was variable in the other species under study, as well as in the most common bacteria known to cause mastitis. Finally, to confirm specificity to the target bacteria, the respective probe sequences were submitted to a BLASTN search. On the 5’ end of each probe a 15mer poli-T sequence was included as well as a thiol group modification. Additionally, a probe that does not hybridize with any target sequence was designed to serve as negative control. Probes’ sequences and characteristics are summarized in Table 2. An additional probe was designed for *S. uberis,* as indicated in Table S1 of the Supplementary Materials.

The DNA single-stranded properties, including melting temperature, guanine and cytosine (GC) content, molecular weight, intermolecular self-complementarity estimation, and intra-molecular hairpin loop formation were calculated using the IDT OligoAnalyzer tool.

### 2.5. Asymmetric PCR Amplification

The asymmetric PCR used a primer ratio of 10:1 (Fw:Rv) and a 50 µL reaction mixture that consisted of 20 µL of 2.5x MasterMix 16S Basic, 1.6 µL of Moltaq 16S, 6 µL of 10 µM, and 1 µM of the forward and reverse primers, respectively, and 2 µL of the template in a total volume of 50 µL. In the negative control, the template was replaced by water. The PCR conditions used include an initial denaturation step at 95 °C for 3 min, followed by 40 cycles of denaturation at 95 °C for 15 s, annealing at 60 °C for 45 s, and an extension at 72 °C for 45 s. In the end, there was a final extension step at 72 °C for 5 min. Amplification was confirmed by agarose gel electrophoresis. 

### 2.6. Detection Assays in the Biochip Platform

Before probe immobilization, the biochips (Figure 1a) went through a cleaning procedure consisting of a 2 h immersion in hot (65 °C) Alconox^®^, followed by thorough rinsing with ultra-pure mili-Q grade water, isopropanol (IPA) and water, and blown dried with a compressed air stream. Finally, they were exposed to an ultraviolet light/ozone plasma for 25 min at 28 mW/cm^2^ at 5 mm separation from the UV lamp inside an UVO cleaner machine from Jelight, (Irvine, CA, USA). The biochips were recycled several times (>10) using this same procedure.

The probes were diluted in the TE buffer to a concentration of 5 µM and immobilized by automatic spotting using a Nano-plotter^TM^ (GeSiM, Germany). Each spot consisted of a sum of 60 droplets, equivalent to a spot volume of ~3 nL. After spotting, the probes were left to immobilize for 1 h inside the spotting machine. Spotted biochips were stored in closed containers at room temperature until further use.

**Figure 2:**
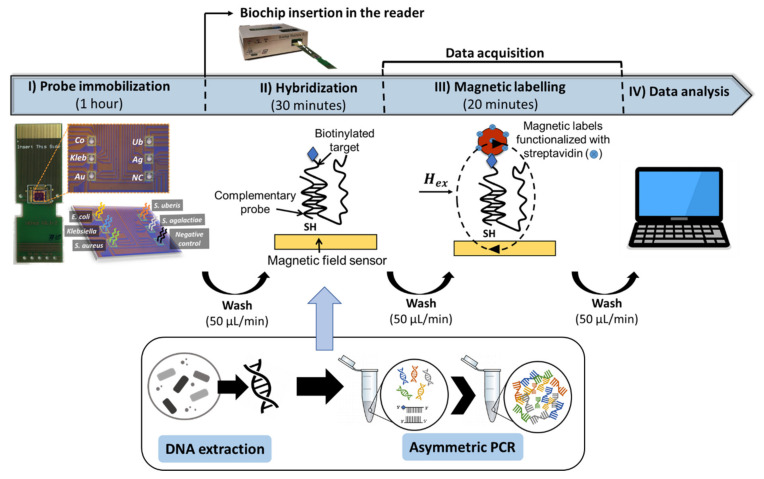
Schematic representation of the main steps involved in a measurement.

The measurement system (Figure S1a) and biochip platform (Figure S1b) are described in greater detail in the Supplementary Materials. The biochip platform was fabricated as described by [23]. After insertion of the spotted biochip into the platform and assemblage of the microfluidic system (Figure 1c) [14], the biochips are rinsed with PB buffer to remove unbound probes, followed by loading of 10 µL of target sample, both pumped into the U-shaped PDMS microchannel with the aid of a syringe pump (NE-300, NEW ERA, NY, USA). The pumping steps were all performed at a flow rate of 50 µL/min. Once the target solution covered the sensing sites, the flow was stopped. Hybridization was left to occur for 30 min at no flow and during the initial 5 min, the current lines surrounding the sensors were activated with a DC field of −60 Oe and an AC field of 20 Oe rms at a frequency of 100 mHz in the electromagnet and a DC current of 30 mA in the current line [21]. After hybridization, unbound target molecules were washed off with PB buffer (phase 1 in Figure 1d)) and 50 µL of MNPs 10× diluted from stock were pumped in and left to settle over the sensors for 20 min (phase 2 in Figure 1d). The unbound particles are then washed off for at least 5 min at continuous flow, until signal stabilization (phases 4 and 5 in Figure 1d, respectively). The main steps necessary for a measurement are represented in Figure 2. In total, data acquisition took about 40 min.

The sensors were biased with a 1 mA DC current. The magnetic drive was set to 35 Oe DC and 13.5 Oe rms AC at 211 Hz. The DC field value was set in the transition to the minimum resistance saturation region of the sensor’s transfer curve, where the sensor response to magnetic labels was maximum, which was in agreement to with Ferreira et al. [24]. For each sensor, a voltage signal was acquired (Figure 1d) and the data was sequentially recorded at a bandwidth of 4 Hz and 2 samples per sensor displayed in the user interface (Figure S2 in Supplementary Materials).

### 2.7. Statistical Analysis

The data were considered to follow a normal distribution. Statistical analysis of the results was performed using one-way analysis of variance (ANOVA) followed by the Tukey–Kramer post hoc test. Differences were considered significant whenever *p* < 0.05.

## 3. Results and Discussion

### 3.1. Asymmetric PCR

**Figure 3:**
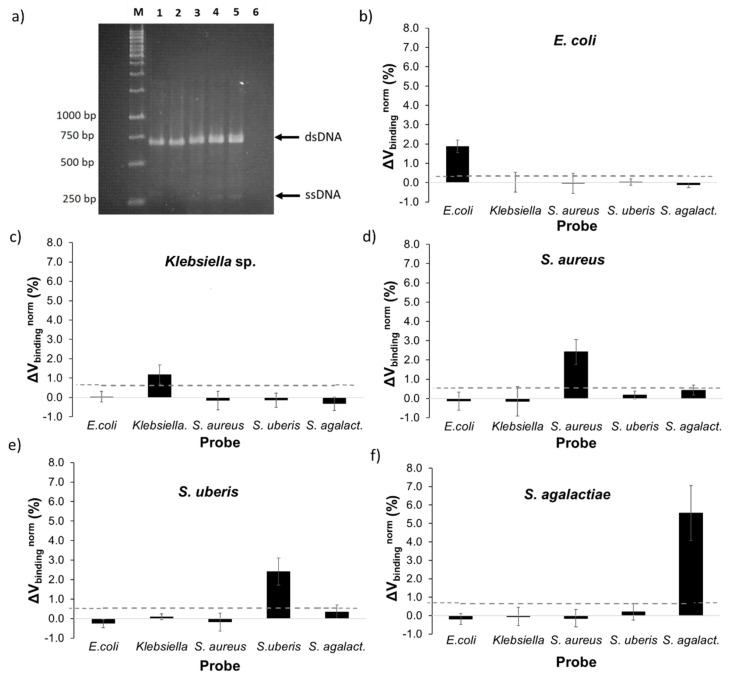
(**a**) Agarose gel electrophoresis of asymmetric polymerase chain reaction (PCR) products obtained from 1 ng/µL of template DNA in the reaction mixture for five mastitic bacterial pathogens. Lane M: 1 kb DNA ladder; lane 1: *E. coli*; lane 2: *Klebsiella* sp.; lane 3: *S. aureus*; lane 4: *S. uberis*; lane 5: *S. agalactiae*; lane 6: negative control (no template DNA). (**b**–**f**) Normalized binding signals obtained for each target amplicon, obtained from asymmetric PCR with 1 ng/µL of initial template DNA in the reaction mixture, against its specific probe and four other unspecific probes. The error bars are standard deviations coming from at least 12 sensors acquired from three (*E. coli*, *S. agalactiae* and *S. uberis* target) or four independent measures (*Klebsiella* sp. and *S. aureus* target) for each target. The dashed line represents the threshold, a minimum value above which a detection signal is considered as significant. The threshold is obtained from the highest signal each probe demonstrated against a non-complementary target. Significant *p*-values (*p*-value <0.0001) were obtained between signals from complementary and non-complementary probes.

All bacterial targets were amplified by asymmetric PCR using one pair of universal primers based on conserved regions of the 16S rRNA gene. Figure 3a presents the agarose gel of PCR amplification products for all the bacteria tested, namely *E. coli* (lane 2), *Klebsiella* sp. (lane 3), *S. aureus* (lane 4), *S. uberis* (lane 5), *S. agalactiae* (lane 6), and the negative control (lane 7). For all targets, two bands were observed at 700 bp and 350 bp-long. As previously reported [25,26,27], the two bands corresponded to the predicted size for dsDNA (700 bp) and ssDNA (350 bp) products from asymmetric PCR. The limiting primer was involved in the production of dsDNA in the first reaction cycle and, when it was fully consumed, started the ssDNA production supported by the forward primer in excess [10]. The efficiency of ssDNA production was significantly weaker in comparison to the dsDNA in accordance with exponential and linear amplification principles [28]. 

Additionally, Gram-positive bacteria exhibited higher product amplification than Gram-negative bacteria. One possible reason could be the difference in GC contents of the bacteria’s genome. While Gram-negative had a GC content between 50–53% [29,30], Gram-positive were reported to be within 33–36% [31,32,33]. This makes the genome of *E. coli* and *Klebsiella* sp. to present a higher melting temperature, which reduced the template amplification efficiency in comparison with *S. aureus*, *S. uberis,* and *S. agalactiae*. It is important to note that a higher efficiency in dsDNA amplification did not necessarily implicate a proportionally higher efficiency in ssDNA production. However, this can only be confirmed by further purification tests.

### 3.2. Detection Assays in the Biochip Platform

Detection assays were performed in the magnetoresistive biochip platform with targets amplified by asymmetric PCR for 1 ng/µL of template DNA. Prior to biochip assays, the specificity of the probes was tested on gold substrates using these PCR products (Figure S3 in Supplementary Materials). The data acquired from each sensor was analyzed in order to obtain the binding signal (ΔV_binding_), which was calculated from the difference between the sensor baseline (V_ac_^sensor^), and the final signal that originated from the bound MPs over the sensor (V_ac_^particles^). To accurately compare the binding signals from different sensors, potentially of different sensitivities, the signals were normalized to the sensor output (V_ac_^sensor^).

In each measurement, one target was tested against the six different probes spotted onto a biochip, including the five specific probes and a negative control probe used as reference signal. At least three biochip measurements were performed for each bacterial target, corresponding to the acquirement of signal of a minimum of 12 sensors. The results obtained are summarized in Figure 3b–f. In each plot, the signal acquired from one of the probes against all five targets was represented, as well as the threshold value above which the detection signal was considered significant. This threshold value was obtained from the highest non-specific signal attained against a non-complementary target, taking into consideration the standard deviation of each signal.

All probes showed specific signals against its complementary target without significant cross reactivity with unspecific probes. Gram-positive probes, as expected due to the apparent higher PCR product quantity, attained higher detection signals than Gram-negative. *S. agalactiae* presented the highest detection signal of 5.6% followed by *S. aureus* and *S. uberis* both with 2.4%, all with threshold values up to 0.7%. Regarding Gram-negative bacteria, *E. coli* showed a reliable detection signal of 1.9 *±* 0.3% and a threshold value of 0.4%. *Klebsiella* sp. detection signal was the lowest (1.2 *±* 0.5%) with a threshold value of 0.6%. 

The results thus confirm that from a single, universal asymmetric PCR protocol, multiplex bacterial detection on the magnetic biochip could be accomplished. Among the five tested bacteria, only *Klebsiella* sp. demonstrated a weak detection signal, which could lead to a difficult identification using this strategy. Therefore, further improvements must be employed, such as the design of a new *Klebsiella* sp. probe, with higher complementarity against the respective DNA target. Additionally, optimization of the asymmetric PCR reaction can also contribute to an increase in detection sensitivity.

### 3.3. S. agalactiae Calibration Curve

**Figure 4:**
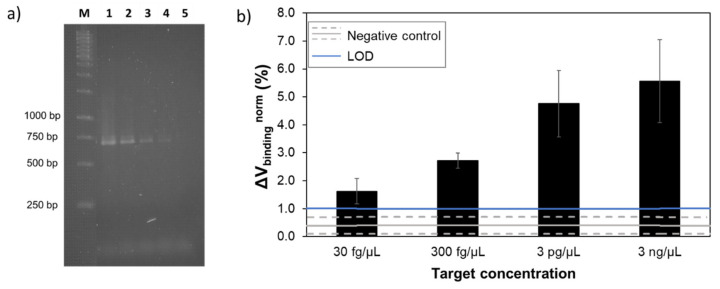
(**a**) 1.5% agarose gel electrophoresis of asymmetric PCR amplicons obtained from template DNA of *S. agalactiae* at different concentrations in the reaction mixture. Lane M: 1 kb DNA ladder; lane 1–4: 3 pg/µL, 300 fg/µL, 30 fg/µL and 3 fg/µL of *S. agalactiae* template DNA, respectively; lane 5: negative control (no template DNA). (**b**) Normalized binding signals obtained from the detection of *S. agalactiae* amplicons resulted from different target concentrations in the PCR mixture. The error bars are standard deviations of the signal of at least 12 sensors, acquired from three independent measurements. The grey line represents the highest signal obtained with *S. agalactiae* against a non-complementary target DNA and dashed lines the corresponding standard deviation. The blue line corresponds to the limit of detection.

To assess the performance of the system in terms of figures of merit, a calibration curve was performed for *S. agalactiae* bacteria, the most promising probe/target pair. Genomic DNA concentrations from 3 pg/µL down to 3 fg/µL were amplified by asymmetric PCR. Amplification was verified by agarose gel electrophoresis (Figure 4a) with all concentrations presenting a visible band for the higher molecular weight (~700 bp). However, no band was visible corresponding to the single stranded product (~350 bp). 

Detection assays were initially performed on gold substrates (Figure S4 in Supplementary Materials) where detection of all concentrations was accomplished. MR biochip calibration curve (Figure 4b) resulted in a semi-quantitative output without a linear behavior due to the end-point amplification step before detection. The lower concentration measured of 30 fg/µL resulted in a detection signal of 1.8 ± 0.7%, well above the negative control, which was 0.4 ± 0.3%. The limit of detection (LOD) was calculated by LOD = NC + 2×SD, where NC is the mean negative control value and SD is the respective standard deviation. The LOD was therefore established at the 1%. After statistical analysis of the measured data, an overall *p*-value <0.0001 was obtained, indicating that the MR signals for different template concentrations were statistically significant. Comparison between the lower target concentration and the negative control, also revealed a *p*-value <0.0001. 

In sum, the detection of *S. agalactiae* target unveiled the potential of the presented system beyond the qualitative output, for the discrimination of different levels of contamination although in a non-linear way. Moreover, the performance of the system was expected to depend on both, PCR and probe hybridization efficiency, not allowing signal prediction for different target concentrations or results extrapolation among different targets. 

In this work, a strategy for the multiplex detection of bacteria was presented using a POC platform. It is shown that using an asymmetric PCR protocol targeting 16S rRNA conserved regions coupled to a magnetic array biochip with species-specific probes, detection of multiple bacteria can be accomplished. The use of asymmetric PCR is a practical and effective method to obtain ssDNA targets. This strategy is simpler than other commonly used methods, such as magnetic beads or Lambda exonuclease [34]. Denaturation of double stranded PCR products by heating is also a common strategy due to its simple and low cost procedure, yet it tends to produce false negative signals in hybridization [35]. Based on *S. agalactiae* calibration curve, the lowest concentration measured was 30 fg/µL of DNA template in the PCR, which corresponds in genome equivalents to ~10^3^ cells/mL. Although detection of 3 fg/µL of DNA template was attained by asymmetric PCR (Figure 4a) and assays performed on gold substrates (Figure S4 in Supplementary Materials), further work is still necessary to achieve successful detection of this concentration using the MR system.

Despite conventional culture being considered the gold standard method for bacterial identification, protocols can take days or weeks to successfully identify bacteria. Recently, next generation sequencing (NGS) of the 16S–23S rRNA encoding region has been proposed for reliable identification of pathogens directly from samples. However, data analysis is laborious, time-consuming, and a specialized technician is required to perform the analysis. Herein, we demonstrated the successful detection of five different bacterial pathogens using a multiplex detection system based on a magnetoresistive biochip portable platform and specific oligonucleotide probes without the need of sequencing, use of bioinformatic tools to analyze the data, or waiting for days for culture results.

One of the main advantages of this system is the controllable and systematic nature of the detection assay. The washing, hybridization, and magnetic labelling conditions are easily reproducible between independent measurements thanks to the microfluidic system. This is an important property particularly for diagnostic devices, where reliability and confidence in the results is essential. Cross-reactivity, which can be a considerable challenge when dealing with closely related bacteria, was overcome with thoroughly designed probes. However, it can also be attributed to the increased stringency of the washing steps imposed by microfluidics and the implementation of a proprietary feature of the MR biochip for local heating during hybridization by on-chip current line actuation. This hypothesis is supported by the results obtained in gold substrates by optical inspection where cross-reactivity was stronger than the obtained in the MR biochip (Figure S3-b in Supplementary Materials). These characteristics make this system a useful tool for mastitis diagnosis, which is still a prevalent problem in the dairy industry. 

## 4. Conclusions

Infectious diseases still represent one of the biggest health burdens worldwide. Bovine mastitis is a common disease in the dairy industry caused by a multitude of bacteria. With bacterial cell culture as the current gold standard for pathogen identification, the diagnosis of mastitic cows is slow and antibiotics are often administered as a preventive measure. As an alternative, DNA-based methods are gaining relevance. However, their integration into practical tools for in situ diagnosis is limited by the difficulty to implement a multiplexed format in microfluidic systems. In this context, we present a simple strategy to attain the detection of multiple bacteria in microfluidic POC devices. 

A magnetoresistive biochip platform combined with the targeting of the 16S rRNA gene was demonstrated for the detection of five bacteria associated to the onset of bovine mastitis. A universal pair of primers and specific oligonucleotide probes were developed based on conserved and interspecific sequence regions of the 16S rRNA gene. Successful amplification of five different bacterial targets was achieved by asymmetric PCR using the designed primers. All probes successfully detected the respective targets without cross-reactivity and a LOD of 10^3^ cell/mL was obtained. These results allowed for the validation of the designed probes as well as the primer set, for the detection of multiple bacteria. Further validation of the technology in mastitis diagnosis will be performed with clinical samples of mastitic milk. Microfluidic sample preparation and nucleic acid amplification units are currently in the prototyping stage to be integrated with the platform. With the fully integrated system, a measurement would be completed within a 4 h timeframe [2]. This technology can also be explored for diagnosis of other clinical applications, including hospital infections and antibiotic resistance genes.

## Figures and Tables

**Table 1:** Sequence, size, guanine and cytosine (GC) content, and melting temperature (T_m_) of a universal pair of primers designed based on the 16S ribosomal RNA gene of five different bacteria.

	Sequence (5′→3′)	Size (bp)	GC%	T_m_ (°C)
Forward primer	GAGTTTGATCMTGGCTCAG	18	50	48
Reverse primer	TRCGCATTTCACCGCTAC	17	53	47

**Table 2:** Sequence, size, GC content, melting temperature (T_m_) and change in free energy of hybridization (ΔG) of the oligonucleotide probes specifically designed to target each bacteria (*E. coli*, *Klebsiella* sp., *S. aureus*, *S. uberis,* and *S. agalactiae*) and the negative control. The melting temperature and Gibbs energy were calculated by the nearest-neighbor model.

Target	Sequence (5′→3′)	Size (bp)	GC%	T_m_ (°C)	ΔG (kcal/mol)
*E. coli*	GAGCAAAGGTATTAACTTTACTCCC	25	40	53.5	−43.90
*Klebsiella* sp.	CACATCCGACTTGACAGA	18	50	51.6	−31.44
*S. aureus*	CACTTTTGAACCATGCGGTTCAAAATATTATCC	33	36.4	58.7	−61.40
*S. uberis*	GAACTATGGTTAAGCCACA	19	42.1	49.5	−33.17
*S. agalactiae*	AACTAACATGTGTTAATCACTCTTATGC	28	32.1	53.8	−44.59
Neg. control	GCCTGGCGATACCGCTGTTA	20	60	57.1	−

## Supplementary Materials

The following are available online at https://www.mdpi.com/1424-8220/20/12/3351/s1. Figure S1: (a) The complete set up of the measurement system includes a syringe pump, used for fluid transport through the microchannel; the biochip platform, where the biochip is inserted and all the electronic circuitry necessary for the measurement is included; and a computer, connected to the biochip platform through a USB connector for data acquisition. (b) Components of the biochip platform: (1) Biochip insertion site; (2) coil for the magnetic drive; (3) battery; (4) noise shielding enclosure for the electronic circuitry; (5) USB connector. Figure S2: Snapshot of the graphic user interface of the platform. Figure S3: (a) Optical microscopic images of a gold substrate (7 × 7 mm^2^) spotted on the Nano-plotter^TM^ with five spots of each specific probe, except for *E. coli* probe, which has a total of 10 spots. (b) Coverage area in percentage of MNPs in gold substrates obtained from the hybridization of each bacterial target. The values corresponding to the detection of complementary targets are highlighted in green. *S. uberis-1* probe was used in this assay. The error bars are standard deviations of the analysis of at least three spots. Table S1: Sequence, size, GC content, melting temperature (T_m_), and change in free energy of hybridization (ΔG) of the two oligonucleotide probes specifically designed to target *S. uberis* (*S. uberis-1* and *S. uberis-2*). The melting temperature and Gibbs energy were calculated by the nearest-neighbor model. Figure S4: Image analysis of MNPs spots obtained from hybridization assays on gold substrates for *S. agalactiae* target amplicons. The percentage of coverage area by MNPs was calculated for each concentration of *S. agalactiae* target. The error bars are standard deviations of the analysis of at least three spots. The black line represents the threshold, which was obtained from the highest signal *S. agalactiae* probe demonstrated against a non-complementary target. Only signals above the threshold are considered significant.

## References

[1] Bradley A.J. (2002). Bovine Mastitis: An Evolving Disease. Vet. J..

[2] Martins S.A.M., Martins V.C., Cardoso F.A., José G., Mónica R., Carla D., Ricardo B., Susana C., Paulo P.F. (2019). Biosensors for On-Farm Diagnosis of Mastitis. Front. Bioeng. Biotechnol..

[3] Park S., Zhang Y., Lin S., Wang T., Yang S. (2011). Advances in microfluidic PCR for point-of-care infectious disease diagnostics. Biotechnol. Adv..

[4] Ashraf A., Imran M. (2018). Diagnosis of bovine mastitis: From laboratory to farm. Trop. Anim. Health Prod..

[5] Llandro J., Palfreyman J.J., Ionescu A., Barnes C.H.W. (2010). Magnetic biosensor technologies for medical applications: A review. Med. Biol. Eng. Comput..

[6] Ferreira J.C., Gomes M.S., Erika C.R., Igor F.C., Edgar F.G., Jamie L.S., Ziyao Z., Fabio S.L. (2018). Comparative analysis of four commercial on-farm culture methods to identify bacteria associated with clinical mastitis in dairy cattle. PLoS ONE.

[7] Romao V.C., Martins S.A.M., Germano J., Cardoso F.A., Cardoso S., Freitas P.P. (2017). Lab-on-Chip Devices: Gaining Ground Losing. ACS Nano.

[8] Henegariu O., Heerema N.A., Dlouhy S.R., Vance G.H., Vogt P.H. (1997). Multiplex PCR: Critical Parameters and Step by Step Protocol. Biotechniques.

[9] Polz M.F., Cavanaugh C.M. (1998). Bias in template-to-product ratios in multitemplate PCR. Appl. Environ. Microbiol..

[10] Srinivasan R., Karaoz U., Marina V., Joanna M.K., Midori K.M., Steve M., Rohan N., Eoin L.B., Susan V.L. (2015). Use of 16S rRNA gene for identification of a broad range of clinically relevant bacterial pathogens. PLoS ONE.

[11] Salipante S.J., Sengupta D.J. (2013). Rapid 16S rRNA Next-Generation Sequencing of Polymicrobial Clinical Samples for Diagnosis of Complex Bacterial Infections. PLoS ONE.

[12] Clifford R.J., Milillo M. (2012). Detection of bacterial 16S rRNA and identification of four clinically important bacteria by real-time PCR. PLoS ONE.

[13] Janda J.M., Abbott S.L. (2007). 16S rRNA gene sequencing for bacterial identification in the diagnostic laboratory: Pluses, perils, and pitfalls. J. Clin. Microbiol..

[14] Martins V.C., Germano J. (2010). Challenges and trends in the development of a magnetoresistive biochip portable platform. J. Magn. Magn. Mater..

[15] Fernandes E., Martins V.C., Nóbregacd C.M., Carvalho C.M., Cardoso F.A., Cardoso S., Dias J., Deng D., Kluskens L.D., Freitas P.P. (2014). A bacteriophage detection tool for viability assessment of Salmonella cells. Biosens. Bioelectron..

[16] Dias T.M., Cardoso F.A. (2016). Implementing a strategy for on-chip detection of cell-free DNA fragments using GMR sensors: A translational application in cancer diagnostics using ALU elements. Anal. Methods..

[17] Li G., Sun S., Wilson R.J., White R.L., Pourmand N., Wang S.X. (2006). Spin valve sensors for ultrasensitive detection of superparamagnetic nanoparticles for biological applications. Sens. Actuators. A Phys..

[18] Lin G., Makarov D., Schmidt O.G. (2015). Strong Ferromagnetically-Coupled Spin-Valve Sensor Devices for Droplet Magnetofluidics. Sensors.

[19] Graham D.L., Ferreira H.A., Freitas P.P. (2004). Magnetoresistive-based biosensors and biochips. Trends Biotechnol..

[20] Freitas P.P., Cardoso F.A. (2012). Spintronic platforms for biomedical applications. Lab. Chip..

[21] Martins V.C., Cardoso F.A., Germano J., Cardoso S., Sousa L., Piedade M. (2009). Femtomolar limit of detection with a magnetoresistive biochip. Biosens. Bioeletron..

[22] Larkin M.A., Blackshields G. (2007). Clustal W and Clustal X version 2.0. Bioinformatics.

[23] Germano J., Martins V.C. (2009). A portable and autonomous magnetic detection platform for biosensing. Sensors.

[24] Ferreira H.A., Feliciano N., Graham D.L., Freitas P.P. (2005). Effect of spin-valve sensor magnetostatic fields on nanobead detection for biochip applications. J. Appl. Phys..

[25] Citartan M., Tang T. (2012). Asymmetric PCR for good quality ssDNA generation towards DNA aptamer production. Songklanakarin J. Sci. Technol..

[26] Veneziano R., Shepherd T.R., Ratanalert S., Bellou L., Tao C., Bathe M. (2018). In vitro synthesis of gene-length single-stranded DNA. Sci. Rep..

[27] Marimuthu C., Tang T.H., Tominaga J., Tan C. (2012). Single-stranded DNA (ssDNA) production in DNA aptamer generation. Analyst.

[28] Gyllensten U.B., Erlich H.A. (2006). Generation of single-stranded DNA by the polymerase chain reaction and its application to direct sequencing of the HLA-DQA locus. Proc. Natl. Acad. Sci. USA.

[29] Touchon M., Hoede C. (2009). Organised Genome Dynamics in the Escherichia coli Species Results in Highly Diverse Adaptive Paths. PLoS Genet..

[30] Hu Y., Wei L., Feng Y., Xie Y., Zong Z. (2019). Klebsiella huaxiensis sp. nov., recovered from human urine. Int. J. Syst. Evol. Microbiol..

[31] Shiroma A., Terabayashi Y. (2015). First Complete Genome Sequences of Staphylococcus aureus subsp. aureus Rosenbach 1884 (DSM 20231T), Determined by PacBio Single-Molecule Real-Time Technology. Genome Announc..

[32] Hossain M., Egan S., Tracey C., Philip N.W., Ray W., James A.L., Richard D.E. (2015). Virulence related sequences; insights provided by comparative genomics of Streptococcus uberis of differing virulence. BMC Genomics.

[33] Kalimuddin S., Chen S.L. (2017). 2015 Epidemic of severe streptococcus agalactiae sequence type 283 infections in Singapore associated with the consumption of raw freshwater fish: A detailed analysis of clinical, epidemiological, and bacterial sequencing data. Clin. Infect. Dis..

[34] Svobodová M., Pinto A., Nadal P., Sullivan C.K.O. (2010). Comparison of different methods for generation of single-stranded DNA for SELEX processes. Anal. Bioanal. Chem..

[35] Gao H., Tao S., Wang D., Zhang C., Ma X.M., Cheng J. (2003). Comparison of Different Methods for Preparing Single Stranded DNA for Oligonucleotide Microarray. Anal. Lett..

