# Supplementary material for: Multiple Bacteria Identification in the Point-of-Care: an Old Method Serving a New Approach
Source: Sensors (Basel). 2020 Jun 12;20(12):3351. doi: 10.3390/s20123351 (PMC7349726; doi:10.3390/s20123351)
Supplement: Supplementary file 1 [file sensors-20-03351-s001.pdf]

Multiple Bacteria Identification in the Point-of-Care: an Old Method Serving a New Approach

Sara Viveiros [1,2], Mónica Rodrigues [3], Débora Albuquerque [1,2], Sofia A. M. Martins [2], Susana Cardoso [1,2] and Verónica C. Martins [2,*]

1 Instituto Superior Técnico, University of Lisbon, Lisboa 1049-001, Portugal; sara.viveiros@tecnico.ulisboa.pt (S.V.); debora.albuquerque@ist.utl.pt (D.A.); susana.freitas@tecnico.ulisboa.pt (S.C.)
2 INESC-MN- Microsystems and Nanotechnologies, Lisboa 1000-029, Portugal; smartins@inesc-mn.pt
3 Centre for Ecology, Evolution and Environmental Changes, Faculdade de Ciências, Universidade de Lisboa, Lisboa 1749-016 , Portugal; marodrigues@fc.ul.pt
* Correspondence: vromao@inesc-mn.pt; Tel. +351 213100237

Supplementary Materials

A) Measurement System

The biochip measurement system includes three main components: the biochip platform, a syringe pump (model NE-300, New Era Pump Systems, Inc.), and a computer for data acquisition (Figure S1a). Figure S1b shows the biochip platform system and its main components: the biochip insertion place; a coil for magnetic drive; a battery; an iron enclosure protecting the platform circuits from interference from external noise sources; and a USB connector, for signal transmission to a laptop computer. Through a graphic user interface (Figure S2), the user defines the biochip type and measurement conditions. All the biochip sensors are then automatically measured and the data is displayed in real-time.

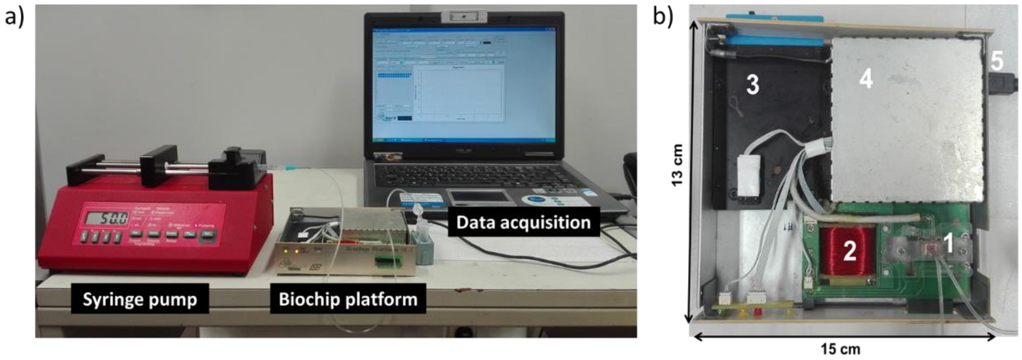

Figure S1. a) The complete set up of the measurement system includes a syringe pump, used for fluid transport through the microchannel; the biochip platform, where the biochip is inserted and all the electronic circuitry necessary for the measurement is included; and a computer, connected to the biochip platform through a USB connector for data acquisition. **b)** Components of the biochip platform: (1) Biochip insertion site; (2) coil for the magnetic drive; (3) battery; (4) noise shielding enclosure for the electronic circuitry; (5) USB connector.

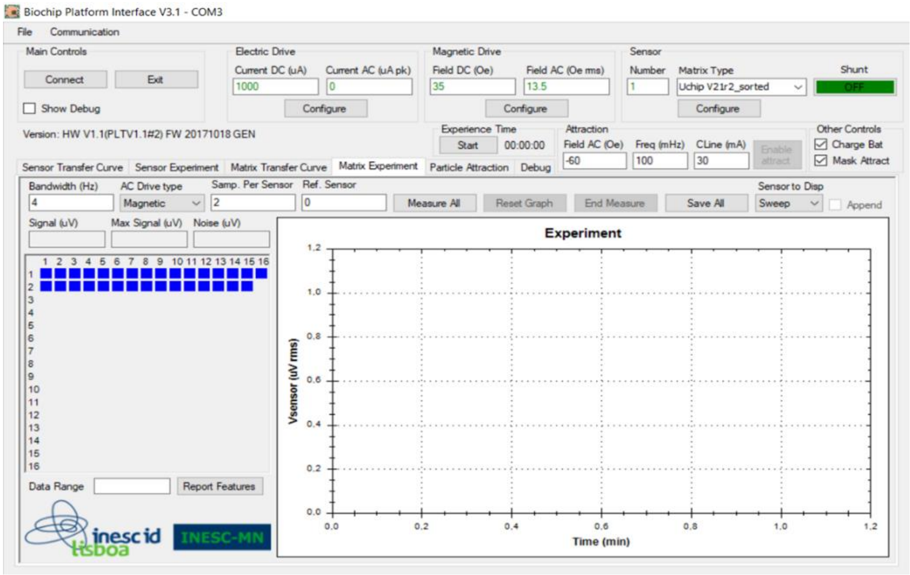

Figure S2. Snapshot of the graphic user interface of the platform.

B) Detection Assays in Gold Substrates

1. Materials and Methods

The detection assays on gold substrates are performed inside a glass petri dish at room temperature in a humid atmosphere to prevent evaporation. Before hybridization, the gold substrates are rinsed with PB buffer to remove excess unbounded probes and the DNA targets are subjected to a denaturation step, consisting of 95 °C for one minute, in a thermocycler. Next, 20 µL of each target (direct PCR product) is added to a spotted substrate (see Figure 1a) and hybridization is left to occur for 1 h. The substrates are then washed in PB buffer to remove unbound target molecules.

A 20 µL suspension of 250 nm streptavidin-coated MNPs diluted 10x from stock (4.9×10^7 particles/ µL) in PB-Tween20 is magnetically concentrated using a DynaMagTM-2 permanent magnet from Life Technologies (Carlsbad, CA, USA) in order to remove the stock solvent. The concentrated MPs are then resuspended in the same volume of PB-Tween20 buffer. The MNP suspension is dispensed over the substrate surface and left to settle down for 20 min. The unbound particles are washed off with PB buffer. The detection of the MNP spots is based on optical microscopy (Leica DM LM) and subsequent analysis on ImageJ software (version 1.52a), where a fixed threshold is applied to delimit the number of pixels corresponding to MNPs. The percentage of surface coverage by MNPs of each spot is automatically calculated and the average value of the five spots corresponding to each probe is obtained, as well as the standard deviation.

2. Results

2.1. Detection Assays with 1 ng/µl of Genomic DNA

Detection assays were initially carried out on gold substrates in order to optically validate the designed probes. Biotinylated amplicons of asymmetric PCR from 1 ng/µL of genomic DNA were added onto functionalized substrates with automated spotted probes (Figure S1a). After hybridization streptavidin-coated MNPs were added for target labelling through biotin-streptavidin binding. Figure S1b shows the results of the ImageJ analysis of the obtained MNP spots. All probes detected their complementary targets. However, at some extent cross reactivity was observed, namely between: *Klebsiella* probe and *E. coli* target, likely because the 16S rRNA gene of these two bacteria is highly conserved; *S. uberis-1* probe and *S. aureus* target; *S. uberis-1* probe, and *S. agalactiae* target. The strongest detection signal was obtained for *S. agalactiae* probe, even surpassing the positive control in intensity.

Due to the considerable cross reactivity that *S. uberis-1* probe was demonstrating against other Gram-positive bacteria, a second probe was designed using SEQUENCHER software (both probes are depicted in Table S1). This probe is significantly smaller (19-mer versus 30-mer of *S. uberis-1*), which increases the specificity for hybridization. The change in free energy of hybridization between each *S. uberis* probe and *S. agalactiae* and *S. aureus* targets was also calculated by the nearest neighbor model, which for *S. uberis-1* resulted in −31.45 and −18.30 kcal/mol, respectively; while *S. uberis-2* probe presents a higher ΔG of −23.79 and −10.71 kcal/mol, respectively. Therefore, theoretically *S. uberis-2* probe should be less prone to cross reactivity with *S. aureus* and *S. agalactiae* targets.

2.2. S. agalactiae calibration curve

Before detection assays in MR biochips, biotinylated amplicons of asymmetric PCR from 3 pg/µL down to 3 fg/µL of *S. agalactiae* genomic DNA were tested on gold substrates. Figure S2 shows the results of the ImageJ analysis of the obtained MNP spots. The specific probe spots resulted in positive signal for all tested concentrations (3 pg/µL, 300 fg/µL, 30 fg/µL and 3 fg/µL) of target amplicons after labelling with MNPs.

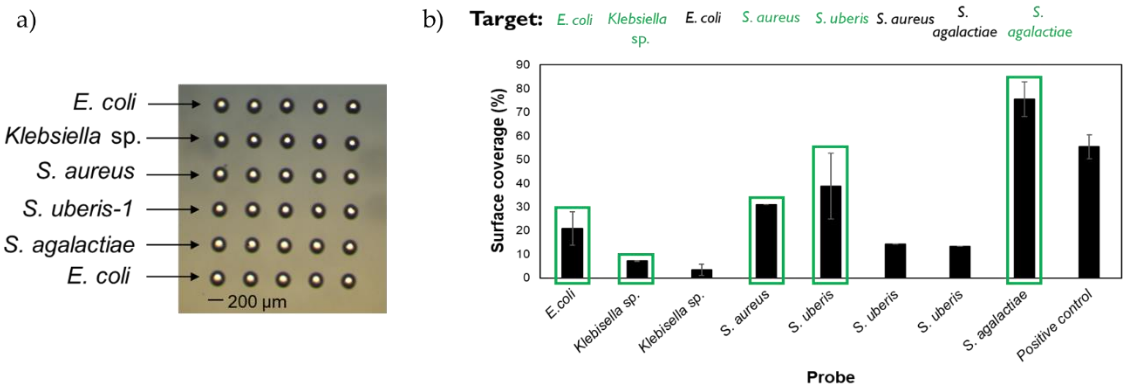

Figure S3. a) Optical microscopic images of a gold substrate (7 × 7 mm^2) spotted on the Nano-plotter™ with five spots of each specific probe, except for *E. coli* probe, which has a total of 10 spots. **b)** Coverage area in percentage of MNPs in gold substrates obtained from the hybridization of each bacterial target. The values corresponding to the detection of complementary targets are highlighted in green. *S. uberis-1* probe was used in this assay. The error bars are standard deviations of the analysis of at least three spots.

Table S1. Sequence, size, GC content, melting temperature (T_m), and change in free energy of hybridization (ΔG) of the two oligonucleotide probes specifically designed to target *S. uberis* (*S. uberis-1* and *S. uberis-2*). The melting temperature and Gibbs energy were calculated by the nearest-neighbor model.

Target	Sequence (5′→3′)	Size (bp)	GC %	T_m(ºC)	ΔG (kcal/mol)
S. uberis-1	GCGTTGCTCGGTCAGGGTTCCCCCCATTGC	30	66.7	70.6	−68.27
S. uberis-2	GAACTATGGTTAAGCCACA	19	42.1	49.5	−33.17

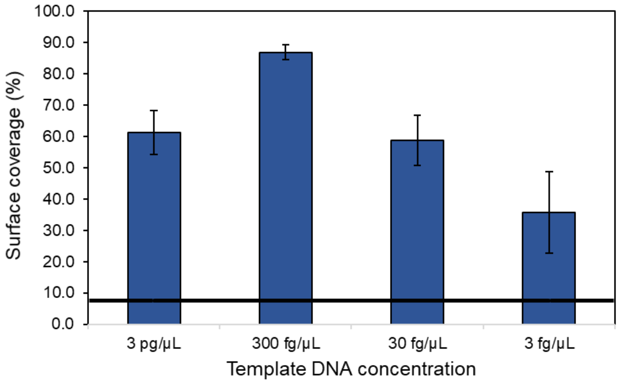

Figure S4. Image analysis of MNPs spots obtained from hybridization assays on gold substrates for *S. agalactiae* target amplicons. The percentage of coverage area by MNPs was calculated for each concentration of *S. agalactiae* target. The error bars are standard deviations of the analysis of at least three spots. The black line represents the threshold, which was obtained from the highest signal *S. agalactiae* probe demonstrated against a non-complementary target. Only signals above the threshold are considered significant.
